# Evidence for a causal link between sepsis and long-term mortality: a systematic review of epidemiologic studies

**DOI:** 10.1186/s13054-016-1276-7

**Published:** 2016-04-13

**Authors:** Manu Shankar-Hari, Michael Ambler, Viyaasan Mahalingasivam, Andrew Jones, Kathryn Rowan, Gordon D. Rubenfeld

**Affiliations:** Guy’s and St Thomas’ NHS Foundation Trust, ICU support Offices, 1st Floor, East Wing, St Thomas’ Hospital, London, SE1 7EH UK; Division of Asthma, Allergy and Lung Biology, Kings College London, London, SE1 9RT UK; Intensive Care National Audit & Research Centre, Napier House, 24 High Holborn, London, WC1V 6AZ UK; Interdepartmental Division of Critical Care Medicine, Sunnybrook Health Sciences Centre, 2075 Bayview Avenue, D5 03, Toronto, Ontario M4N 3M5 Canada

**Keywords:** Sepsis, Mortality, Causality, Bias, Confounding factors (epidemiology)

## Abstract

**Background:**

In addition to acute hospital mortality, sepsis is associated with higher risk of death following hospital discharge. We assessed the strength of epidemiological evidence supporting a causal link between sepsis and mortality after hospital discharge by systematically evaluating the available literature for strength of association, bias, and techniques to address confounding.

**Methods:**

We searched Medline and Embase using the following ‘mp’ terms, MESH headings and combinations thereof - sepsis, septic shock, septicemia, outcome. Studies published since 1992 where one-year post-acute mortality in adult survivors of acute sepsis could be calculated were included. Two authors independently selected studies and extracted data using predefined criteria and data extraction forms to assess risk of bias, confounding, and causality. The difference in proportion between cumulative one-year mortality and acute mortality was defined as post-acute mortality. Meta-analysis was done by sepsis definition categories with post-acute mortality as the primary outcome.

**Results:**

The literature search identified 11,156 records, of which 59 studies met our inclusion criteria and 43 studies reported post-acute mortality. In patients who survived an index sepsis admission, the post-acute mortality was 16.1 % (95 % CI 14.1, 18.1 %) with significant heterogeneity (*p* < 0.001), on random effects meta-analysis. In studies reporting non-sepsis control arm comparisons, sepsis was not consistently associated with a higher hazard ratio for post-acute mortality. The additional hazard associated with sepsis was greatest when compared to the general population. Older age, male sex, and presence of comorbidities were commonly reported independent predictors of post-acute mortality in sepsis survivors, challenging the causality relationship. Sensitivity analyses for post-acute mortality were consistent with primary analysis.

**Conclusions:**

Epidemiologic criteria for a causal relationship between sepsis and post-acute mortality were not consistently observed. Additional epidemiologic studies with recent patient level data that address the pre-illness trajectory, confounding, and varying control groups are needed to estimate sepsis-attributable additional risk and modifiable risk factors to design interventional trials.

**Electronic supplementary material:**

The online version of this article (doi:10.1186/s13054-016-1276-7) contains supplementary material, which is available to authorized users.

## Background

Sepsis [1] is a global health care challenge [2]. Although acute mortality from sepsis in adults is improving [3], nearly a third of sepsis survivors are readmitted to hospital within 30 days of discharge [4–6], have a three-fold greater infection risk [7], and have persistently elevated markers of inflammation [8]. In a systematic review that included patients from both critical care (ICU) and non-ICU settings, sepsis was associated with impaired quality of life and increased long-term mortality [9].

However, outcomes after critical illness reflect a complex interplay between patient demographics, comorbidity, risk factors for critical illness, treatments in the ICU, and critical illness itself [10, 11]. Therefore, the poor outcomes observed in survivors of sepsis may be caused by sepsis or they may simply reflect events that would have occurred in these patients had they not become septic or had they been admitted to the hospital for reasons other than sepsis [10–14].

It is an important question, as a more comprehensive understanding of the causes and mechanisms of the post-ICU syndrome might inform more effective interventions. If post-sepsis long-term outcomes are primarily driven by the trajectory of pre-morbid conditions, then interventions targeted at complications attributed to critical illness may not be effective. Approaches to this question might take many forms including an analysis of biologic plausibility from animal studies of sepsis. Randomized trials that involve inducing sepsis are, of course, impossible in humans. Therefore we focused on observational studies of sepsis or cohorts derived from randomized trials. This study aims to systematically review the existing epidemiologic literature to specifically assess how a causal link between sepsis and mortality after hospital discharge is addressed, by evaluating the available literature for strength of association, impact of bias, and techniques to address confounding, to determine if increased long-term mortality reported after sepsis is caused by sepsis.

## Methods

### Approach

The standards for causal inference from observed associations in epidemiology are well-established [15–17]. These include associations that are strong and independent of bias or confounding, consistent across studies, specific to the exposure, demonstrate an increasing risk of the outcome with higher levels of exposure (dose-response), and are biologically plausible [14, 18]. We evaluated the extent to which studies fulfilled these criteria using the following framework. First, we separated the cumulative long-term mortality in to its components of acute and post-acute mortality. The causal effects of sepsis on acute mortality are well-described. As cumulative long-term mortality incorporates acute mortality, an evaluation of the long-term effects of sepsis must isolate its unique effects on post-acute mortality from its effects on acute mortality. Second, the study design and analytic approach to confounding and bias were measured. This included whether patients were lost to follow up and the use of restriction, matching, stratification, and regression. We evaluated which variables the authors used as potential confounders. Third, as design and analytic approaches to confounding may be insufficient, we looked for the type and number of control populations included to minimize bias in the comparison and to clarify whether the outcomes following sepsis exposure were specific or merely a reflection of survival from critical illness. Finally, we assessed whether studies evaluated a dose-response relationship between sepsis and post-acute mortality, with the hypothesis that more severe sepsis, for example, septic shock, should be associated with higher post-acute mortality within a study and there should be an inverse relationship between acute mortality and post-acute mortality between studies.

### Information sources

A systematic review of non-randomized and randomized clinical studies indexed within the Medline and the Embase databases were performed using the Ovid platform. Search terms included the following ‘mp’ terms, medical subject headings (MESH) and combinations thereof - sepsis, septic shock, septicemia, outcome, quality of life, cohort studies, and randomized controlled trials. The following limits were applied: humans, English language and publications since 1992. Subject headings were *exploded* and mapped to the appropriate MeSH. The search was restricted to English language articles published in or after 1992, when the first consensus definitions were introduced [19]. The full electronic search strategy for Medline is presented in electronic supplementary material (Additional file 1: Table S1).

### Eligibility criteria for full text review

We included studies in adult patients reporting an episode of sepsis defined using either the 1992 [19] or the 2003 [20] consensus definitions with the following mandatory criteria: (1) the studies must report all-cause one-year or longer mortality and (2) must report the consensus definition components that could be assessed. In our search strategy we included studies reporting quality of life or cognitive outcomes on post-discharge follow up and initial full text review to identify studies that reported long-term mortality as one of the secondary outcomes. To generate a more homogenous sepsis population, we excluded studies exclusively reporting pediatric patients, patients with retroviral disease or cancer, other specific cohorts such as immune-compromised patients, and obstetric cohorts.

### Study selection for evidence synthesis

Two authors (MA and VM), using predefined inclusion criteria based on review of the titles and abstracts, performed independent assessment of studies identified within the literature search. Consensus was reached on the inclusion of studies after independent review of the studies (by MSH) and by mutual agreement of the reviewers. In order to avoid including the duplicate data where multiple articles were found that presented data from the same cohort of patients, the most relevant article was chosen by consensus (MA and MSH). To obtain the full text of the included studies, the authors were contacted if it was not possible to obtain from existing databases (n = 1) [21].

### Data collection process and items

Two authors (MA and MSH) extracted data from the included studies and issues of uncertainty were resolved by consensus. From each of the included studies we extracted the following data to explore generic and study-specific quality checklists. The generic quality checklist included variables to inform a modified Newcastle-Ottawa score (NOS): study years (recruitment), country, single or multi-center, study design, number of patients, duration of follow up, evidence of selection bias at enrollment, proportion of patients lost during follow up, loss during follow up explained and key outcomes reported (Additional file 1: Table S2). The study-specific quality checklist included variables to assess (1) baseline risk of death (age, sex, comorbidity score or index, or pre-sepsis functional status, or comorbidity type); (2) acute illness risk of death (severity of illness score or a surrogate); (3) description of sepsis (definition, sepsis categories); (4) approach to bias; (5) approach to confounding; (6) separation of mortality endpoints to address post-acute mortality; and (7) use of non-sepsis control groups for comparisons.

### Outcome definitions

**Fig. 1:**
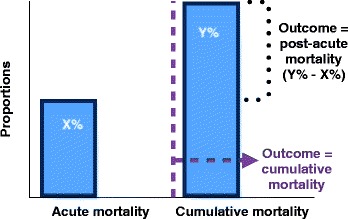
Approach to the study question. Post-acute mortality was estimated as the difference between one-year mortality and acute mortality, to address the study questions as described (see “Methods”, “Approach”). We assessed whether the studies used cumulative mortality or post-acute mortality in regression models. This was done to identify risk factors that are associated with post-acute mortality, which will help future researchers delineate modifiable risk factors. This approach helps to generate a summary estimate of post-acute mortality and also explores the relationship between acute mortality and post-acute mortality at cohort level

In this study, acute mortality refers to hospital mortality during the index sepsis admission episode (28-day or ICU mortality was used when hospital mortality was not reported). Cumulative one-year mortality refers to the total reported mortality at one year. The primary study outcome was post-acute mortality, which we defined as the difference between cumulative and acute mortality (Fig. 1).

### Description of bias assessment

In addition to the bias assessments described in the generic quality checklist (Additional file 1: Table S2), descriptions of setting and data source were used to assess the accuracy of exposure (sepsis) and information bias. To assess the risk of bias from loss to follow up, we collected data on completeness of follow up and how the primary outcome of interest (post-acute mortality) was ascertained. We coded the risk of ascertainment bias as low and follow up as complete, if the studies used either national/regional databases or by direct contact with the patient/family.

### Description of confounding assessment

The study design and analytic methods used to address confounding in studies were categorized as restriction, matching, stratification, and regression. Restriction as a method to reduce confounding was defined either as use of specific sepsis/infection populations or as use of any additional criteria to specifically influence the relationship between sepsis and post-acute mortality (for example, when only patients with no comorbidities were included in an analysis). Matching was coded as yes if performed either at study design or during analysis. Stratified analysis was coded as yes if the studies report mortality stratified on predictor variables. Any use of multivariate regression to examine mortality or survival time was coded. We evaluated regression models separately depending on whether they used post-acute, or cumulative mortality as the outcome variable. The sepsis case definition was categorized as either consensus based, or modified from consensus definition, or other.

### Statistics

We calculated the difference in proportion between cumulative one-year mortality and acute mortality. This post-acute mortality rate and the cohort size were used to estimate the number of post-acute deaths. Random effects meta-analysis was done to generate summary post-acute mortality estimates. A contour-enhanced funnel (confunnel) plot was used to assess publication bias [22]. The distributions of statistical significance in the confunnel plot are derived from the Wald statistic for the effect estimate of each study, with asymmetry implying potential publication bias. Egger’s test for small-study effects was done, which regress the standard normal deviate of the study effect estimate against its standard error. The dose-response effect refers to an increase in the risk of adverse outcomes with increase in severity of sepsis. If a cohort has high acute mortality, the survivors may or may not have higher post-acute mortality. An aaplot was used to estimate this dose-response relationship between acute mortality and post-acute mortality [23]. Finally, we performed two sensitivity analyses using random effects meta-analysis of post-acute mortality by including only studies with an NOS score ≥5, as a surrogate for high study quality and by the acute-mortality time point reported. All analyses were done using Stata/MP 13.1 StataCorp College Station, TX, USA.

## Results

### Study selection

**Fig. 2:**
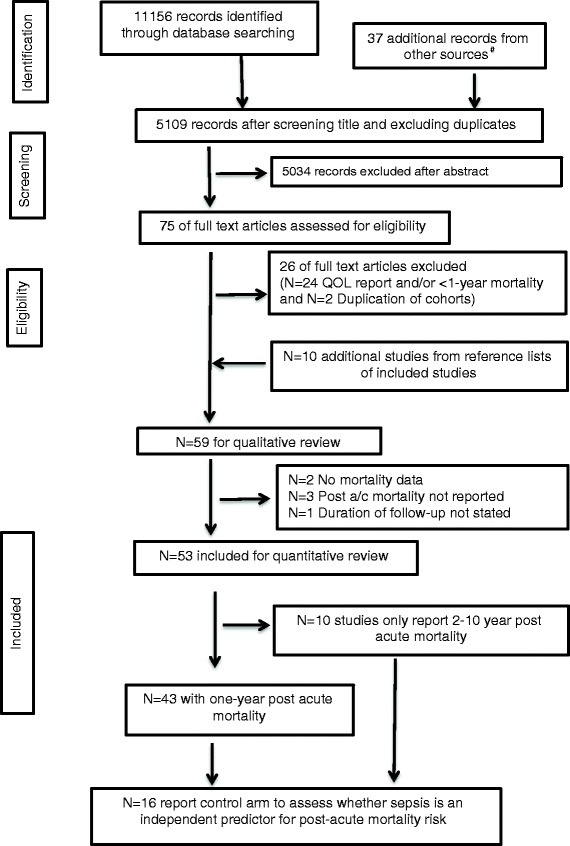
Flow diagram showing the literature search and results. ^#^Non-duplicate articles were identified from the references list of two review articles [9, 10]. *QOL* quality of life. A/c refers to *acute*

The literature search identified 11,156 records. Following exclusion of duplicates, and title screening, there were 5,109 abstracts to screen. Following abstract screening, 75 articles met the criteria for full text evaluation. We excluded a further 26 articles reporting quality of life and/or follow-up mortality for less than one year (n = 24) and 2 studies using data from the same cohort. Ten additional studies were identified from the reference scan of the 49 studies that were included from full text review. Thus, 59 studies were included for qualitative review. Amongst these two studies did not report mortality data [24, 25], post-acute mortality could not be estimated from three further studies [26–28], and one study did not report the duration of follow up [21]. Thus, 53 studies were included for quantitative review, of which 43 studies contained sufficient data to estimate one-year post-acute mortality. Ten studies that report 2-year to 10-year mortality were excluded from quantitative review [11, 29–33], but were included for information in answering other questions. Only 16 studies included control arms so that the causal effects of sepsis on post-acute mortality could be assessed [7, 25, 30–43]. (Fig. 2; Table 1; Additional file 1: Table S3-S4).

**Table 1:** Assessment of study quality, risk of bias, and confounding

Methodological assessment summary (N=59 studies)	%*
1. Exposure definition (sepsis)	
i) Consensus [7, 8, 28–32, 35, 37, 39, 40, 44, 45, 47–50, 63–74]	49 %
ii) Claims/ICD codes [11, 24–26, 38, 41, 58, 75–77]	14 %
iii) Pathogen-plus (either positive blood culture or pathogen specified) [27, 34, 36, 46, 53, 78–81]	20 %
iv) Pneumonia [33, 40, 42, 43, 52, 82–85]	15 %
v) Peritonitis [21, 51, 86]	5 %
2. Ascertainment of exposure	
i) Claims/ICD codes data	13 %
ii) Randomized controlled trial cohorts [44, 45, 63, 66, 69, 70]	10 %
iii) Non-interventional study cohorts	75 %
3. Selection of the control cohort	
i) Drawn from ICU patients without sepsis	
a. Matched [35]	2 %
b. Not matched [7, 25, 30–32, 37, 41]	12 %
ii) Drawn from hospitalized infected patients (not ICU)	
a. Matched	
b. Not matched [30, 41]	3 %
iii) Drawn from hospitalized non-infected patients (not ICU)	
a. Matched [36]	2 %
b. Not matched [30, 34, 38–41]	10 %
iv) Population controls	
a. Matched [30, 33, 37, 41–43]	10 %
b. Not matched [40, 41]	3 %
v) No control cohorts/no controls for mortality comparison	73 %
4. Comparability of cohorts on the basis of design or analysis	
i) Regression models to adjust for confounders	
a. Regression models in studies reporting control population	
- Post-acute mortality [7, 25, 30, 33–37, 39, 42]	16 %
- Cumulative mortality	
- Stratified analysis for post-acute mortality [32, 40, 42, 43]	5 %
- Non-mortality outcome models [31, 38, 41]	5 %
b. Regression models using in studies with no controls	
- Post-acute mortality	22 %
- Cumulative mortality	20 %
- No mortality model	31 %
ii) Studies reporting sepsis dose-response [39, 46, 49, 52, 53]	9 %
5. Assessment of post-acute mortality	
i) Record linkage with national or regional databases or outcome assessed by contact with patient or relatives	90 %
ii) No description	10 %
6. Adequacy of follow up	
i) Complete follow up, all participants accounted for	22 %
ii) Loss to follow up unlikely to introduce bias (<20 % loss, or >20 % but those lost described and unlikely to be different from those followed)	61 %
iii) Follow up <80 % and no description of those lost	3 %
iv) No statement	14 %
7. Report both acute and post-acute mortality (or that information can be determined from the reported data)	
i) At one year	72.9 %
ii) Between 2 and 10 years [11, 29–32]	8.5 %
iii) No mortality data/post-acute mortality not estimable/follow-up time unclear [21, 24–28]	10.2 %

*Reported proportions (%) corrected to nearest whole number. Study quality and risk of bias was assessed on selection, comparability, and reported outcomes using the Newcastle Ottawa Score checklist (Additional file 1: Table S2). Sepsis case definition evaluates the representativeness of the cohort and ascertainment of exposure by assessing concordance with the sepsis definitions to the definition of Bone et al. [19] or Levy et al. [20]. Studies reporting data from randomized controlled trials (RCTs) are likely to have a lower score as non-sepsis controls are not evaluated

### Post-acute mortality

**Fig. 3:**
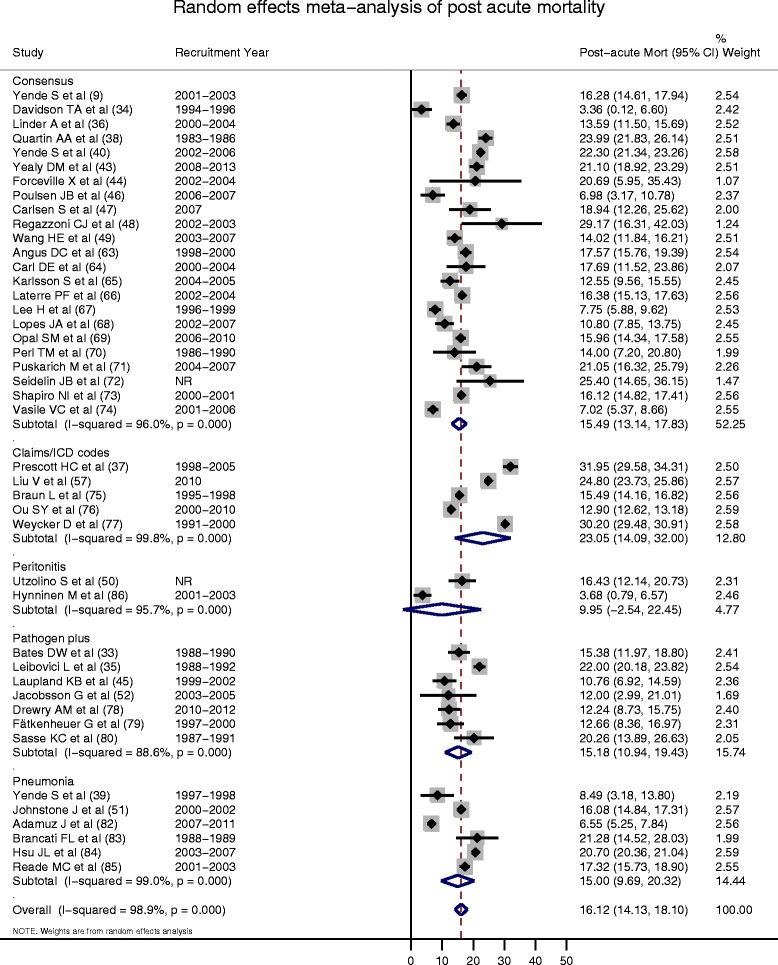
Random effect meta-analysis of post-acute mortality. First author, reference number and cohort recruitment year for each study are shown. *X axis* indicates mortality proportions. *NR* not reported, *Recruitment Year* refers to recruitment window reported in studies

**Fig. 4:**
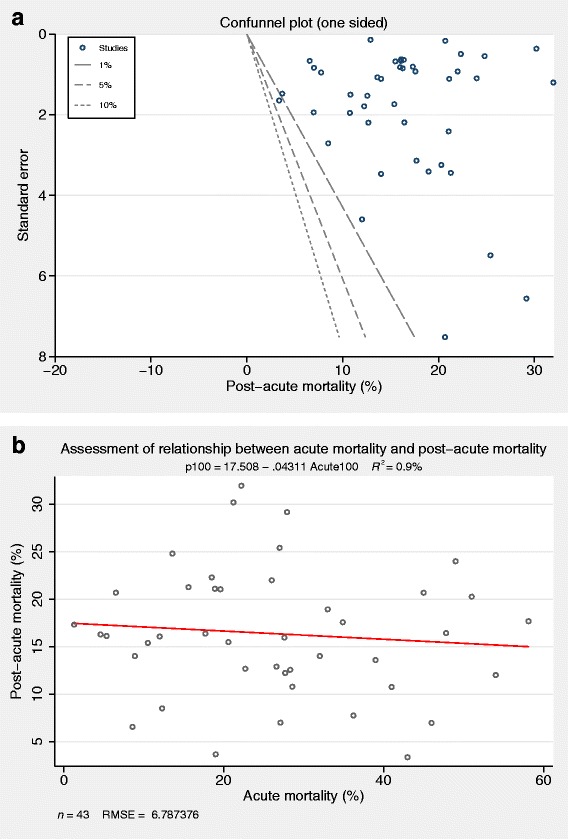
**a** One-sided contour-enhanced funnel (confunnel) plot. The figure either implies potential publication bias or consistency in results (implying causality) across the published studies identified by this systematic review. The confunnel plot adds contours of statistical significance to the standard funnel plot and assesses whether the areas where studies are potentially missing correspond to areas of low statistical significance, the assumption being that studies that do not attain statistical significance boundaries are less likely to be published. **b** Post-acute mortality versus acute mortality with linear fit superimposed and assessed between study dose-response effects. The equation, R-square statistics of the fit, the sample size and root mean square standard error (RMSE)(s) are also shown (referred to as aaplot and designed by Nicholas J. Cox)

The mean (95 % CI) one-year post-acute mortality estimated from 43 studies using random effects meta-analysis was 16.1 % (14.1, 18.1 %) with significant heterogeneity (*I*^2^ = 98.9 %; *p* < 0.001) (Fig. 3). The one-sided confunnel plot shows asymmetry, which could be interpreted either as implying potential publication bias or as consistent results across studies supporting causality (Fig. 4a). There were no statistically significant small-study effects (Egger’s test *p* = 0.883; Additional file 1: Table S5). Studies that included patients with higher mortality in acute sepsis did not demonstrate lower mortality in the post-acute period from a shift in the timing of death (Fig. 4b). Most studies report patient cohorts prior to 2005 (Fig. 3). In sensitivity analyses, the post-acute mortality (reported as mean (95 % CI)) were consistent with primary analysis (in high quality studies (n = 33), mean =16.3 % (13.6–19.0 %); *I*^2^ = 98.6 %; *p* < 0.001, in studies reporting hospital mortality (n = 23), mean = 16.6 % (13.5–19.6 %); *I*^2^ = 99.0 %; *p* < 0.001, and in studies reporting 28- or 30-day mortality (n = 20), mean = 15.8 % (14.1–17.4 %); *I*^2^ = 93.3 %; *p* < 0.001).

### Bias assessment

The risk of bias in the included studies was variable, with asymmetric NOS distribution (Table 1; Additional file 1: Table S3; Additional file 1: Figure S1). Consensus criteria were the most commonly used case definitions. Case identification was often retrospective, thus in these cases there was potential for information bias secondary to accuracy of exposure. RCTs are likely to have the least risk of information/exposure ascertainment bias. National or regional databases (with or without contact with the family) were used by most studies to ascertain post-acute mortality, implying this would not be a major contributor to bias. Overall risk of bias and quality assessed using modified NOS score variables highlighted 10 studies with high risk of bias/poor quality (NOS score <5; Additional file 1: Figure S1 and Additional file 1: Table S3).

### Confounding assessment

Confounding in the relationship between sepsis and post-acute mortality potentially occurs when risk factors or confounding variables are distributed unequally between survivors and non-survivors. Studies without control groups cannot answer the causal question, but inform the reasons for the variability observed in studies.

The definitions for patient inclusion criteria in the studies could be categorized into five groups (pneumonia, consensus sepsis based definitions and modifications thereof, consensus sepsis definitions but pathogen identification mandated in the inclusion criteria, claims/ICD codes for sepsis, and peritonitis cohorts (Table 1). There was significant heterogeneity in post-acute mortality within these categories (*p* < 0.001). In five studies restricted to patients with septic shock, the post-acute mortality varied between 7.0 % and 21.1 % [44–48]. Similar variations in the relationship between sepsis and post-acute mortality were observed in studies limited to elderly populations [11, 24, 25, 38, 40, 41, 49], a southern US cohort aged 45 years or older [50], male patients [39], obese patients [51], and in a separate analysis restricted to a previously healthy population [37]. This implies, the determinants of the relationship between sepsis and post-acute mortality are unclear, thus, key restriction variables need to be ascertained.

### Studies reporting controls

**Fig. 5:**
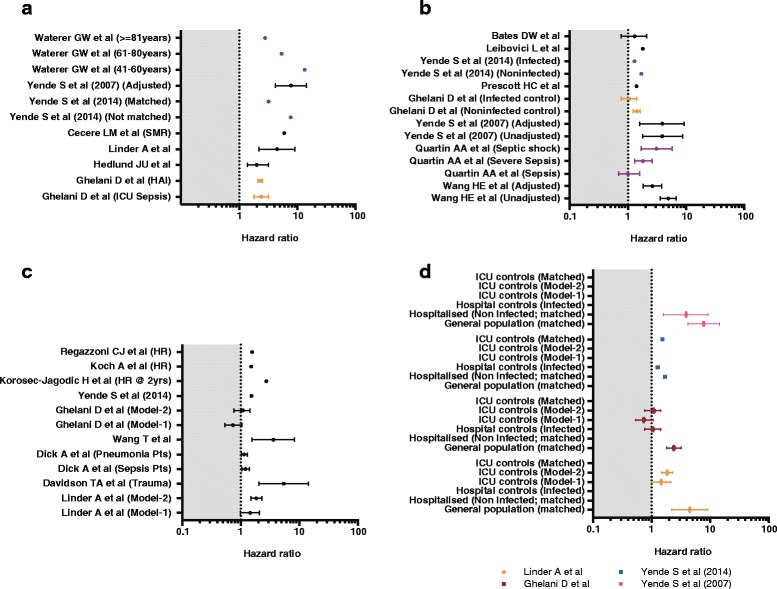
Additional post-acute hazard with sepsis. To illustrate the differences in additional hazard with sepsis, all four sub-graphs were generated with the same scale on the *x axis. Dashed line* at *hazard ratio 1* is the reference line; *shaded area* shows the range between *0* and *1*. If the same study reported risk-adjusted and unadjusted hazard ratios, these are presented together to highlight confounding. If only proportions are reported, they are presented as *dots. Confidence intervals* of hazard ratios are shown when reported in studies. **a** Additional hazard when compared to general population controls. **b** Additional hazard when compared to hospitalized controls. Hazard ratios associated with single episode of pneumonia are reported for Yende S et al. [40]. **c** Additional hazard when compared to critically ill controls. **d** Confounding from studies reporting multiple controls. In Linder et al, the additional risk of sepsis compared to critically ill controls (Model-1) and cardiovascular system (CVS) surgical controls (Model-2) between 1 and 5 years [37]. In Ghelani et al, the additional risk of sepsis compared to critically ill controls (Model-1) and hospital infected controls (Model-2) [30]. In all the graphs, the hazard ratios are either reported by the study or estimated by comparing the sepsis outcomes to reported control populations. *HAI* healthcare-associated infection, *SMR* standardized mortality ratio, *HR @ 2 yrs* refers to hazard ratio at 2 years, *Model-1*, *Model-2* different risk-adjusted models reported in studies. Additional details are provided in Additional file 1: Table S6

There were 16 studies that included control groups to evaluate the effects of sepsis on post-acute mortality; 7 studies used the general population [30, 33, 37, 40–43], 13 used hospitalized controls (ICU, infected patients, and non-infected patients) [7, 25, 30–32, 34–41], and 4 studies used both [30, 37, 40, 41]. From these studies we evaluated whether sepsis is an independent risk factor for post-acute deaths and what other independent predictor variables were associated with post-acute mortality in sepsis patients (Additional file 1: Table S3, S4 and S6; Fig. 5a-d)

### Studies reporting general population controls

In age- and sex-matched life-table data comparisons, post-acute mortality in pneumonia cohorts was two [43] to six times [33] higher than in the general population. Interestingly, when the age-, sex- and race-matched comparisons were stratified by comorbid status, the significant post-acute mortality risk seen in young patients with pneumonia remained in those with comorbidity and was no longer statistically significant in those without comorbidities, implying that the crude effects were likely attributable to age and comorbidity [42]. Sepsis cohorts requiring ICU care also have two- to five-fold higher risk of post-acute death than the general population [30, 36, 37, 39, 41].

### Studies reporting hospital controls

The unadjusted post-acute mortality for severe sepsis when compared to hospitalized controls was no longer significantly different when adjusted for confounders such as age and comorbidities [30]. Amongst eleven studies with non-sepsis controls, six studies include sepsis as an exposure within regression models to evaluate the independent effect of sepsis on post-acute mortality. Sepsis was an independent predictor in four studies [25, 35, 37, 39]. Sepsis ceases to be an independent predictor of post-acute mortality as the comparator group severity of illness increases from hospitalized non-infected to ICU non-sepsis (Fig. 5b-d) [7, 25, 30–41]. The common non-sepsis predictor variables in the regression models from these eleven studies were age (n = 8) and comorbidity (n = 8).

### Dose-response effect of sepsis on post-acute mortality

We observed an inconsistent sepsis–post-acute mortality dose-response effect in studies. The post-acute risk of death increases with worsening pneumonia [52] and with sepsis severity [39, 49]. Unexpectedly, septic shock was associated with a significant reduction in post-acute hazard ratio (0.77 (0.68–0.86); *p* < 0.001) [46], whilst sepsis was associated with higher post-acute mortality than severe sepsis (17.8 % vs. 14.0 %) [53] again highlighting the importance of *baseline-risk* factors on post-acute deaths amongst sepsis survivors.

## Discussion

The main finding of this systematic review is that an additional 16.1 % of deaths among patients admitted with acute sepsis occur between hospital discharge and one year in patients who survive an index admission with sepsis (post-acute mortality). Post-acute mortality was identified in studies using national databases and loss to follow up at one year was low, implying a lower risk of ascertainment and information bias. In studies that assess whether sepsis is an independent predictor of post-acute mortality, the magnitude of additional risk attributable to sepsis was inconsistent. Even in studies with low risk of bias, the limited reporting of sepsis subgroups precludes generating summary assessments. Common predictors of post-acute mortality were age and comorbidity. Studies that assessed the effect of sepsis on post-acute mortality with multiple control populations highlight the potential for confounding in the studies that either do not use controls or report unadjusted data for the relationship between sepsis and post-acute mortality (Fig. 5). In studies that report control populations, comorbidity, male sex, baseline functional status and acute severity of illness were highlighted as potential factors influencing the relationship between sepsis and post-acute mortality.

The key strengths of our systematic review include limiting the study population to adult sepsis cohorts and limiting the outcome to mortality. We separated cumulative long-term mortality into its two components, which address importantly different hypotheses. A customized NOS checklist enabled us to assess potential for bias in the ascertainment of exposure and outcome in addition to how included studies addressed confounding. We also used accepted criteria to assess causality in these studies [14, 18].

There are limitations to this systematic review. To address our study question, we defined long-term outcome as one-year mortality and excluded studies reporting shorter post-discharge outcomes. As this was a systematic review we were limited to the available studies, many of which included patient cohorts studied prior to 2005. It is possible that recent trends in acute mortality from sepsis might mean that appropriately performed studies would uncover a persuasive causal link between sepsis and post-acute mortality. This could occur if patients who would have died from sepsis in past years now survive to hospital discharge and now succumb to sepsis-specific morbidity in the post-acute phase. A similar issue limits the analysis of the dose-response effect of sepsis on post-acute mortality. If more severe forms of sepsis increase acute mortality, any effect on post-acute mortality will not be seen. The studies had limited access to comorbidity and many factors were not accounted for, including the trajectory of prior hospitalizations, nutritional status, discharge location, and family support. However, given the direction of the effects of confounding it is likely that more accurate recording of confounders would further blunt any independent associations between sepsis and post-acute mortality. There is related literature outside the remit of this systematic review, reporting the independent impact of acute respiratory distress syndrome (ARDS) on long-term outcomes [54, 55]. However, pneumonia and extra-pulmonary sepsis account for nearly 75 % of the ARDS cohort in the recently published LUNG-SAFE observational study [56], implying potential utility of our review to this population. Finally, although our analysis argues against outlier effects from small studies, we cannot exclude the possibility that larger cohorts could identify a smaller effect of sepsis on post-acute mortality.

A systematic review of long-term outcomes from sepsis has been publised previously [9], and whilst there are similarities to our review there are a number of key differences. First, we defined and evaluated post-acute mortality at one year. Second, we focused on evaluating the potential causality relationship between sepsis and post-acute mortality. Third, we specifically assessed risk of bias in studies, beyond generic quality checklists. Fourth, to answer the causality question, we identified key confounders, risk of confounding, and methods used to address confounding in studies. Fifth, by fixing the outcome of interest as one-year post-acute mortality, we were able to perform random effects meta-analysis and explore publication bias whilst taking study quality into account [22].

Our systematic review highlights the need for a more coherent approach to understand the true magnitude of the additional, independent, and potentially modifiable impact of sepsis on post-acute mortality. First, the baseline risk variables could be categorized into fixed risk factors (e.g., age, sex, and race), and modifiable risk factors (e.g., comorbidities). Different comorbidities may have a different impact on outcomes and comorbidities themselves may progress following acute insults [10, 13]. The health status trajectory prior to the acute illness trajectory is an important determinant that needs to be accounted for in future studies [24]. Thus young, previously healthy population without comorbidities is an important subset to study in addition to the more common older population with pre-existing comorbidities. Second, index sepsis admission is often followed by readmission(s), which may impact on one-year survival [57]. This readmission is often related to infection [4, 7, 58], which highlights the need for basic science and translational  research into the contributing mechanisms. Third, the duration of modifiable higher risk needs to be explored. There may be a therapeutic window to potentially deliver enhanced post-acute care and a novel window for trials of new interventions. Frailty occurs in patients with sepsis and in sepsis survivors [24, 59, 60]. Therefore, future studies should aim to document the influence of the pre-illness trajectory and post-acute survival time on the progression of underlying comorbidities and frailty [10, 13]. Researchers need to consider whether cause-specific mortality may add useful information in long-term outcome studies of acute illnesses like sepsis. These approaches that we propose for research into sepsis-related post-acute mortality have similarities with the post-intensive care syndrome stakeholder priorities [61]. Finally, sepsis and septic shock has been recently redefined [1, 62], which also prompts reassessment of the post-acute mortality and mobidity epidemiology using more recent patient cohorts.

## Conclusions

The available literature is of insufficient quality to sustain the hypothesis that sepsis exerts an independent and potentially causal effect on post-acute mortality. Inferences about a causal link between sepsis and post-acute mortality is based on a few studies that have inadequately adjusted for confounders in the relationship between acute illness and longer-term survival, such as age, effect of comorbidity, pre-acute illness trajectory, and functional status at hospital discharge.

## Key messages

- Post-acute mortality in sepsis survivors is common; however, causality and the magnitude of this relationship is uncertain
- Acute mortality from sepsis has improved with time. Most studies in this systematic review report patient cohorts recruited before the year 2005 and report additional one-year risk of death following sepsis. Only a minority of included studies report control cohorts and explicitly assess sepsis-specific additional risk
- Our systematic review thus highlights the need for well-conducted studies using more recent datasets to identify the independent (and modifiable) predictors of post-acute mortality in sepsis survivors

## Additional file

Additional file 1: Table S1. Search strategy. **Table S2.** Modified Newcastle Ottawa Score (NOS). **Table S3.** Description of studies included in the systematic review. **Table S4.** Baseline risk, acute illness risk, mortality by sepsis category, and outcomes in sepsis patients. **Table S5.** Egger’s Test for small-study effects. Note: data input format theta se_theta assumed. **Table S6.** Confounding and causality assessment from studies reporting control arms. **Figure S1.** Distribution of customized Newcastle Ottawa Score in the studies identified by the systematic review. (DOC 361 kb)

## Abbreviations

ARDS: acute respiratory distress syndrome
confunnel plot: contour-enhanced funnel plot
NOS: Newcastle-Ottawa score
QOL: quality of life
RCT: randomized controlled trial
