# Supplementary material for: Evidence for a causal link between sepsis and long-term mortality: a systematic review of epidemiologic studies
Source: Crit Care. 2016 Apr 13;20:101. doi: 10.1186/s13054-016-1276-7 (PMC4831092; doi:10.1186/s13054-016-1276-7)

# ESM File

**Supplemental Digital Content – Table S1: Search strategy**

**Supplemental Digital Content – Table S2: Modified Newcastle Ottawa Score [NOS]:**

This a modified Newcastle-Ottawa Score for our study question. We assessed seven domains of study design, each with up to five mutually exclusive categories. Of those categories, some were deemed to be of ‘high’ quality and therefore scored a point. Total points for each study across the eight domains were summed to give the final NOS score. This tool was modified in order to assess the quality of studies with non-randomised design. Application of this tool to studies in our review produces a single numerical score. The tool is not customized for assessing randomized control trials. The NOS judges retrospective studies to be of lower quality than prospective studies. There were inconsistencies between included studies regarding whether the authors considered their study to be retrospective or prospective design despite considerable methodological similarities. For the purpose of scoring studies, we followed the authors’ own assessment of whether the design was prospective or retrospective, where such a comment was made in the text, otherwise the design was judged by consensus [MA and MSH]

* Indicates the content that scores a point.

**Supplemental Digital Content – Table S3: Description of studies included in the systematic review**

Based on sepsis case definition, the cohorts are categorized as described. Assessment of confounding is described in the main manuscript. The outcome used in the logistic regression model and the statistically significant predictor variables are summarized. In studies where a regression model is not reported, the variables shown to be statistically significant in stratified analyses are reported. Variables associated with non-mortality outcomes are not presented.

**Supplemental Digital Content – Table S4:** **Baseline risk, acute illness risk, mortality by sepsis category and outcomes in sepsis patients**

Age is presented as reported in studies either mean (standard deviation) or median (inter quartile range). Proportion of males in the stud cohort is shown. Co-morbidity is summarized as presented by studies either as proportions by organ system, or as diseases, or as composite scores. Acute illness risk is summarized either as severity of illness scores (APACHE II or SAPS scores) or as organ dysfunction scores as reported by studies. Mortality by sepsis category assesses dose response and is shown where reported by studies. Acute mortality and cumulative mortality at one-year when reported are presented.

**Supplemental Digital Content – Table S5: Egger’s Test for small study effects. Note: data input format theta se_theta assumed.**

**Supplemental Digital Content – Table S6: Confounding and causality assessment from studies reporting control arms**

**Supplemental Digital Content – Figure S1: Distribution of customized Newcastle Ottawa Score in the studies identified by the systematic review**

**Table S1: Search strategy:**

Database: Ovid MEDLINE(R) 1946 to June 2015

Searched via OvidSP interface: 30^th^ June 2015

1. sepsis.mp. or Sepsis/

2. septic shock.mp. or Shock, Septic/

3. septic?emia.mp.

4. or/1-3

5. mortality.mp. or Hospital Mortality/ or Mortality/

6. outcome$.mp. or Fatal Outcome/ or "Outcome Assessment (Health Care)"/ or "Outcome and Process Assessment (Health Care)"/ or Treatment Outcome/

7. Cognition/ or <cognitive outcome>.mp. or Neuropsychological Tests/

8. prognosis.mp. or Prognosis/

9. quality of life.mp. or "Quality of Life"/

10. or/5-9

11. cohort studies.mp. or Cohort Studies/

12. clinical trial.mp. or Clinical Trial/

13. Random Allocation/ or random$.mp.

14. 20

15. or/11-14

16. 4 and 10

17. 15 and 16

18. limit 17 to english language

19. limit 18 to humans

20. limit 19 to "all adult (19 plus years)"

21. limit 20 to yr="1992 -Current”

**Table S2: Modified Newcastle Ottawa Scale**

1. Sepsis Definition + additional criteria:

i) Truly representative of the consensus (1992 or 2001)*

ii) Not representative of above

iii) No description of derivation of the cohort

2. Ascertainment of exposure (severe sepsis in ICU setting):

i) Claims from insurance, medicare or Medicaid schemes*

ii) From a RCT or prospectively identified cohort*

iii) National surveillance program*

iv) Retrospectively identified cohort

3. Selection of the control cohort:

i) Drawn from critically ill patients without a diagnosis of sepsis*

ii) Drawn from not critically ill but satisfying sepsis definition above*

iii) Age- and/or sex-matched hospital controls*

iv) No control cohort

4. Comparability of cohorts on the basis of design or analysis:

i) Matched study design*

ii) Regression models to adjust for confounders*

iii) No clear identifiers for comparability

5. Assessment of outcome (mortality):

i) Record linkage with national or regional databases*

ii) Outcome assessed by contact with patient or relatives*

iii) No description

6. Adequacy of follow-up:

i) Complete follow-up, all participants accounted for*

ii) Loss to follow-up unlikely to introduce bias (<20% loss, or >20% but those lost described and unlikely to be different from those followed)*

iii) Follow-up <80% and no description of those lost

iv) No statement

7. Report both early (i.e. 28 or 30d/ICU/hospital mortality) and mortality post-discharge

**Table S3: Description of studies included in the systematic review**

| **Author (Enrolment period)**  **Setting** | **N**  **&**  **Follow up time** | **Location**  **Single/Multi**  **Cohort type** | **Sepsis case definition** | **Confounder Assessment in studies** | | | | **Independent predictor variables in regression models or stratified analysis for mortality** | **Bias** |
| --- | --- | --- | --- | --- | --- | --- | --- | --- | --- |
|  |  |  |  | **Restriction** | **Matching** | **Stratified analysis**  **reported** | **Mortality modelled in regression model** |  | **NOS** |
| Adamuz J et al[82]  2007-2011  ICU and non-ICU | N=1405  1-year | Spain  Single centre  Non-interventional | Pneumonia | Yes | No | No | Post acute mortality | COPD, DM, Cancer, Dementia, Rehospitalisation, Nursing home | 5 |
| Al Thaqafi A et al [27]  (2002-2009  ICU and non-ICU | N-258  1-year | Saudi Arabia  Single centre  Non-interventional | Candida BSI | Yes | No | Yes | No mortality model | No data | 1 |
| Angus DC et al[63]  (1998-2000)  ICU | N=1690  Maximum 3.6 years | International;  Multicentre  RCT; | Modified Consensus  Criteria | Yes | No | Yes | No mortality model | Higher early deaths in older; pre-illness dependent state and more severe acute illness | 6 |
| Bates DW et al [34]  1988 – 1991  ICU and non-ICU | N=439  3 year | USA  Single centre  Non-interventional | Bacteraemia | Yes | Yes | No | Post acute mortality | Severity of illness  None of the septic shock survived 1-year following hospital discharge | 8 |
| Brancati FL et al[83]  1988-1989  Non-ICU | N=141  2-years | USA  Multicentre  Non-interventional | Pneumonia | Yes | No | Yes | Post acute mortality | Comorbidity; Haematocrit<35% | 4 |
| Braun L et al[75]  (1995-1999)  ICU and non-ICU | N=2834  1 years | USA;  Multicentre  Non-interventional | ICD-9 | No | No | No | No mortality model | Age >=35 years using univariate analysis | 6 |
| Carl DE at al[64]  (2000-2004)  ICU | N=147  1 years | USA;  Single-centre  Non-interventional | Consensus  Criteria | Yes | No | Yes | Cumulative mortality | Survival at one year was associated with younger patients, lower initial HR; higher initial temperature; starting dialysis with a BUN <100 mg/dL | 3 |
| Carlsen S et al[48]  (2007)  ICU | N=132  1 years | Denmark;  Multicentre  Non-interventional | Consensus  Criteria | Yes | No | Yes | Cumulative mortality | Resuscitation fluid volume was not associated with post-acute mortality | 6 |
| Cecere LM et al. [33]  1994-1996  ICU and non-ICU | N=552  Up to 6 years | USA  Single centre  Non-interventional | Pneumonia | Yes | No | Yes | Post acute mortality | Age>65years; HCAP: High risk pneumonia (PSI III and IV); HIV infection; CVS disease; Immunosuppressant drugs | 6 |
| Chao PW et al. [26]  2000-2010  ICU and non-ICU | N=31,070  10 years | Taiwan  Multicentre  Non-RCT | Consensus  Criteria | No | Yes | Yes | Post acute mortality | Control arms were propensity matched for intervention assessment | 4 |
| Cuthbertson B H et al[29] (2003)  ICU | N=439  5 years | Scotland  Multicentre  Non-interventional | Consensus  Criteria | No | No | No | No mortality model | No mortality modelling | 6 |
| Davidson TA [35]et al  1994-1996  ICU | N-258  Median= 753 days | USA  Single centre  Non-interventional | Consensus criteria | No | Yes | Yes | Post acute mortality | Age and co-morbidity | 7 |
| Dick A et al [25]  2001-2007  ICU | N=17,537  1 year pre and 5 year post index admission | USA  National dataset  Non-interventional | Elderly with pneumonia or sepsis (ICD codes) | Yes | No | Yes | Cumulative mortality | Age, male, insurance, sepsis and pneumonia | 6 |
| Drewry AM et al [78]  2010-2012  ICU | N=335  1-year | USA  Single centre  Non-interventional | Consensus  Criteria | Yes | No | Yes | Cumulative mortality | Lymphopenia | 1 |
| Fatkenheuer G et al[79]  1997 – 2000  ICU and non-ICU | N=229  1-year | Cologne  Multi centre  Non-Interventional | Staphylococcus Aureus bacteraemia | Yes | No | Yes | Cumulative mortality | Age, unknown source, pneumonia, malignant disease | 4 |
| Forceville X et al[45] (2002-2004)  ICU | N=60  1 year | France  Multicentre  RCT | Modified Consensus  Criteria | No | No | No | No mortality model | Not applicable | 4 |
| Ghelani D et al[30] (1993-1999)  ICU and non-ICU | N=224  4.8 years | Australia;  Single centre  Non-interventional | Consensus  Criteria | No | Yes | Yes | Post acute mortality | Age; male sex; Charlston co-morbidity score; severity of illness; positive blood culture | 7 |
| Haraldsen P et al[21]  1982-1996  ICU | N=210  Not reported | Sweden  Single centre  Non-interventional | Abdominal sepsis | Yes | No | No | No mortality model | No data | 2 |
| Hedlund JU et al[43]  1987  Non-ICU | N=277  Average 31 month follow-up | Sweden  Single centre  Non-interventional | Pneumonia | Yes | No | Yes | Cumulative mortality | Corticosteroid treatment | 5 |
| Hsu JL et al[84]  2003-2007  ICU and non-ICU | N=55,963  One-year | USA  Multicentre  Non-interventional | Pneumonia | Yes | No | Yes | Post acute mortality | Health-care associated pneumonia | 4 |
| Hynninen M et al[86]  2001 – 2003  ICU and non-ICU | N=163  1-year | Helsinki, Finland  Single centre  Non-interventional | Secondary peritonitis | Yes | No | Yes | Cumulative mortality | APACHEII score, Baseline functional status, sepsis category | 4 |
| Iwashyna TJ et al[11] (1996-2008)  ICU and non-ICU | N=516  Median=0.92 year | USA  Multicentre  Non-interventional | Claims definitions based on Consensus Criteria; >=65 years | Yes | No | Yes | Non mortality outcomes | Pre-sepsis baseline characteristics and trajectory of decline with elderly conditions | 6 |
| Iwashyna TJ et al [24]  1998-2006  ICU and non-ICU | N=>1,000,000  5 years | USA;  Multicentre  Non-interventional | Consensus  Criteria | Yes | Yes | Yes | No mortality model | 3-year sepsis survivorship changes due to incidence cases | 6 |
| Jacobsson G et al[53] (2003-2005)  ICU and non-ICU | N=50  3 years | Sweden;  Single centre  Non-interventional | MSSA bacteraemia | Yes | No | Yes | Post acute mortality | Age, comorbidity, nosocomial infection and severe sepsis | 6 |
| Johnstone J et al[52]  2000-2002  ICU and non-ICU | N=3415  Up to 6 years | Canada  Multicentre  Non-interventional | Pneumonia | Yes | No | Yes | Cumulative mortality | Older age, male sex and higher Pneumonia severity index scores | 5 |
| Karlsson S et al[65] (2004-2005)  ICU | N=470  2 years | Finland;  Multicentre  Non-interventional | Consensus  Criteria | No | No | No | No mortality model | Age on univariate analysis | 6 |
| Koch A et al[31]  (Not stated)  ICU | N=164 sepsis  3 years | Germany  Single centre  Non-interventional | Consensus criteria | No | No | No | No mortality model | Study evaluated the utility of measuring asymmetric dimethyl arginine levels | 4 |
| Korosec-Jagodic H et al[32]  (2003)  ICU | N=66  2 years | Slovenia;  Single centre  Non-interventional | Consensus  Criteria | No | No | No | No mortality model | No data | 7 |
| Laterre P F et al[66] (2002-2004)  ICU | N=3376  1 year | International;  Multicentre  RCT | Modified Consensus  Criteria | Yes | No | Yes | Cumulative mortality | Not applicable | 6 |
| Laupland KB et al[46] (1999-2002)  ICU | N=251  683 days (381- 1029) | Canada;  Multicentre  Non-interventional | Modified Consensus  Criteria  (Positive blood culture) | Yes | No | Yes | Post acute mortality | Hazard Ratio<1 for Severity of illness (APACHE II Score and TISS score)  Hazard Ratio>1 for Age; Bacteraemia; surgical diagnosis | 5 |
| Lee H et al[67]  (1996-1999)  ICU | N=787  3 years | Canada;  Multicentre  Non-interventional | Modified Consensus  Criteria (Prowess) | No | No | No | No mortality model | Age on univariate anlaysis | 5 |
| Leibovici L et al[36]  1988 – 1992  ICU and non-ICU | N=1991  4-years | Israel  Single centre  Non-interventional | Evidence of infection and modified Consensus Criteria | Yes | Yes | Yes | Post acute mortality | Age, functional class, malignancy, neutropenia, heart failure, serum albumin and creatinine levels; CVS disease; Inappropriate empiric antibiotics | 7 |
| Linder A et al[37]  2000 – 2004  ICU | N=2289  Mean=10.4 years | Canada  Single centre  Non-interventional | Consensus criteria | No | Yes | Yes | Post acute mortality | Age, co-morbidity, heart failure | 8 |
| Liu V et al[58]  ICU and non-ICU | N=6344  1-year | USA  Multi centre  Non interventional | ICD codes based on consensus definitions | No | No | Yes | Cumulative mortality | No mortality specific covariates reported; pre-sepsis health status was a significant association with survival and heath care use | 5 |
| Lopes JA et al[68]  (2002-2007)  ICU | N=426  2 years | Portugal;  Single centre  Non-interventional | Modified Consensus  Criteria (Acute kidney injury) | Yes | No | Yes | Post acute mortality | Age; Acute kidney injury | 5 |
| Opal SM et al[69]  (2006-2010)  ICU | N=1961  1 year | International;  Multicentre  RCT | Consensus  Criteria | Yes | No | No | No mortality model | Not applicable | 5 |
| Ou SY et al[76]  2000-2010  ICU and non-ICU | N=55584  Up to 10 years | Taiwan  Multicentre  Non-RCT | ICD-9 | Yes | Yes | Yes | Cumulative mortality | Unadjusted analysis - Age, sex, Heart failure, Diabetes, Statin use | 4 |
| Perl et al[70]  (1986-1990)  ICU and non-ICU | N=100  6 years | USA;  Single centre  RCT | Modified Consensus  Criteria (gram negative sepsis) | Yes | Yes | Yes | Post acute mortality | Severity of underlying illness and McCabe and Jackson class; co-morbidities; vasopressors; ARDS | 5 |
| Poulsen JB et al[47] (2006-2007)  ICU | N=172  1 year | Denmark;  Single centre  Non-interventional | Consensus  Criteria (septic shock) | Yes | Yes | No | No mortality model | No data | 6 |
| Prescott HC et al[38]  1998-2005  ICU and non-ICU | N=1493  2-years | USA  Multi centre  Non-interventional | Claims data codes | No | Yes | Yes | No mortality model | No data | 8 |
| Puskarich M et al [71]  2004 – 2007  ED | N=285  1-year | USA  Single centre  Interventional (non-RCT) | Consensus Criteria | No | No | No | Cumulative mortality | Hazard ratio<1 EGDT  Hazard ratio>1 SOFA | 6 |
| Quartin et al[39]  (1983-1986)  ICU and non-ICU | N=898  8 years | USA;  Multicentre  RCT | Consensus  Criteria | No | No | No | Cumulative mortality | Severity of inflammation; gastrointestinal co-morbidity;  Septic shock | 8 |
| Reade MC et al. [85]  2001 – 2003  ICU and non-ICU | N=2183  1-year | USA  Multicentre  Non-Interventional | Pneumonia | Yes | No | Yes | Cumulative mortality | Age, Co-morbidity; Smoking; Men | 5 |
| Regazzoni CJ et al[49] (2002-2003)  ICU | N=137  1 year | Argentina;  Multicentre  Non-interventional | Consensus  Criteria | No | No | Yes | Post acute mortality | Severity of illness; poor functional status; respiratory, neurological and hepatic failure; cognitive and physical disability | 5 |
| Sasse et al[80]  (1987-1991)  ICU | N=153  1 year | USA;  Single centre  Non-interventional | Modified Consensus  Criteria  (Positive blood culture) | Yes | No | No | Post acute mortality | Severity of illness (in the first month); Comorbidities (HIV; malignancies); certain pathogens | 5 |
| Seidelin JB et al[72]  (Not stated)  ICU | N=63  1 year | Denmark;  Single centre  Non-interventional | Consensus  Criteria | No | No | No | No mortality model | No data | 6 |
| Shapiro NI et al [73]  2000-2001  ED | N=3,102  1-year | USA  Single centre  Non-interventional | Modified consensus definition (clinical decision to obtain blood culture) | No | No | Yes | Post acute mortality | MEDS score variables derived risk | 5 |
| Utzolino A et al[51]  (Not stated)  ICU | N=253  5 years | Germany  Single centre  Non-interventional | Secondary peritonitis | Yes | No | Yes | No mortality model | No data | 2 |
| Vasile VC et al[74]  2001-2006  ICU | N=926  3-year | USA  Single centre  Non-interventional | Consensus criteria + troponin measurement | Yes | No | Yes | Post acute mortality | Age, sex | 5 |
| Wang T et al[7]  2001-2002  ICU | N=78  1-year | USA  Single centre  Non-interventional | Consensus Criteria | No | Yes | Yes | Post acute mortality | Presence of sepsis; age; neurological dysfunction | 6 |
| Wang HE at al [50]  2003-2007  ICU and non-ICU | N=970 sepsis  6-year | USA  Multicentre  Non-interventional | Consensus criteria | No | No | Yes | Post acute mortality | Male, Health status, comorbidities, admission to ICU | 7 |
| Waterer GW et al[42]  1998-2001  ICU and non-ICU | N=404  Up to 4 years | USA  Single centre  Non-interventional | Pneumonia | Yes | No | Yes | Cumulative mortality | Age; CVD; CVS disease; Altered mental status; Haematocrit<35%; Increasing blood glucose | 5 |
| Weycker D et al[77] (1991-2000)  ICU and non-ICU | n=16,019  ~8 years | USA;  Multicentre  Non-interventional | Consensus  Criteria + bacteraemia/fungaemia | No | No | Yes | No mortality model | Stratified analysis shows age, organ dysfunction, comorbidity as significant predictors | 6 |
| Yaw LK et al[81]  1997-2007  ICU and non-ICU | N=583  Up to 14 years | Australia  Single centre  Non-interventional | Staphylococcus bacteraemia | Yes | No | Yes | No mortality model | No data | 5 |
| Yealy DM et al[44]  Up to 2013  ICU and ED | N=1341  Septic shock  1-years | USA Multicentre  RCT | Consensus criteria Lact>4mmol/L | Yes | No | No | No mortality model | Not applicable | 4 |
| Yende S et al[8]  2001-2003  ICU and non-ICU | N=1799  1-year | USA Multicentre  Non-interventional | Pneumonia +/- Sepsis Consensus Definitions | No | No | Yes | Post acute mortality | Age, sex, race, co-morbidity, location after hospital discharge, IL-6, IL-10 (all stratified analyses) | 5 |
| Yende S et al[40]  1997-1998  ICU and non-ICU | N=106 with CAP  5-years | USA  Multicentre  Non-interventional | Pneumonia | Yes | No | Yes | Post acute mortality | Comorbidities – Cancer, CVA, and heart failure; Fracture; pneumonia | 6 |
| Yende S et al[41]  2002- 2006  ICU and non-ICU | N=4,179 severe sepsis  1-year | USA  Multicentre  Non-interventional | Consensus Definitions | No | Yes | Yes | No mortality model | Outcome modelled was CVS events after severe sepsis | 6 |
| Zhang K et al[28]  2003 – 2008  ICU | N=479  3 years | China  Multicentre  Non-interventional | Consensus criteria | No | No | Yes | No mortality model | Only HRQOL comparison | 5 |

**Table S4: Baseline risk, acute illness risk, mortality by sepsis category and outcomes in sepsis patients**

| Author | Baseline risk | | | Acute illness risk | Mortality by sepsis category  :a/c - LT | Acute mortality | Cumulative one-year mortality |
| --- | --- | --- | --- | --- | --- | --- | --- |
|  | Age  mean (sd) | Male  (%) | Co-morbidity  (%) | Severity score |  |  |  |
| Adamuz J et al[82] | 69 (53-78)  80 (72-85) | 65.7% | COPD 29.1%  DM 25.3%  CVS disease 16.6%  Cancer 8.3%  CVD 11.1%  Dementia 5.5%  Liver disease 6.1%  CKD 9.9% | 9.6% admitted to ICU | NR | 8.6% | 15.2% |
| Al Thaqafi A et al [27] | Paediatric and adults | 53.2% | Cancer 43.8%  Neutropenia 29.5%  DM 29.0%  Dialysis 10.9% | 53.4% admitted to ICU | NR | NR | 55.2% |
| Angus DC et al[63] | 60.5 (16.8) | 57.0% | Major co-morbidity 17.5% | 24.8 (7.8) ^a^ | NR | 34.9% | 42.8% |
| Bates DW et al [34] | Overall not reported | 48.3% | Major co-morbidity 13.4% | NR | NR | 10.5% | 26.0% |
| Brancati FL et al[83] | Population categorised by age | 76.0% | Mild 13.0%  Moderate 54.0%  Severe 33.0% | NR | NR | 16.0% | 36.9% |
| Braun L et al[75] | 50.0 (NR) | 53.1% | ICD-9 circulatory 76%  ICD-9 respiratory 73% | 70.2%>=1 organ dysfunction | NR | 20.6% | 36.1% |
| Carl DE at al[64] | 52.2 (15.3)  55.8 (12.0) | 67.3% | Malignancy 20.4%  Cirrhosis 30.6%  DM 30.0%  COPD 21.1%  Heart failure 23.1% | Mechanical ventilation 90.5%;  24.5 (NR) ^a^ | NR | 58.1% | 76.4% |
| Carlsen S et al[48] | 60 (17) | 53.0% | NR | 52 (16) ^s^  52 (18) ^s^ | All septic shock | 33.0% | 52.0% |
| Cecere LM et al. [33] | 47.3 (16.4) | 77.0% | Immune 21.0%  Cancer 8.0%  Cirrhosis 4%  CKD 2.0%  CVS disease 24.0%  CVD 6%  DM 6%  COPD 24.0%  Prev pneumonia 9% | 36% were classed as Pneumonia Severity index IV or V | NR | 12.5% | - |
| Chao PW et al. [26] | 69.1 (14.8)  66.8 (16.7) | 59.5%  54.7% | Charlson index>=1  97.1% and 94.5% | 62.3% and 59.9%>=1 organ dysfunction | NR | NR | 26.4% |
| Cuthbertson B H et al[29] | 58 (45-67) | 53.0% | APACHE II co-morbidity 0.2 (0.5) | 23.0 (17.0-28.0) ^a^ | NR | 43.0% | NR |
| Davidson TA [35]et al | Sepsis specific data not extractable |  |  |  |  | 43.0% | 47.0% |
| Dick A et al [25] (sepsis) | 74.1 (NR) | 50.1% | Co-morbidity present in 94.25% | NR | NR | NR | NR |
| Drewry AM et al [78] | 60.8 (15.3)  66.3 (12.8) | 57.0% | Heart failure 34.3%  CAD 32.5%  CVD 18.2% DM 40.6%  CKD 27.2%  Liver 17.6%  COPD 35.2% | 17.1 (5.5) ^a^  22.5 (6.6) ^a^ | NR | 27.7% | 40.0% |
| Fatkenheuer G et al[79] | 56.1 (17.9) | 66.8% | CVS disease 30.6%  Cancer 28.8% DM 22.7%  CKD 17.5%  Liver 14.0% | NR | NR | 22.7% | 35.8% |
| Forceville X et al[45] | 69 (12)  66 (14) | 63.3% | Hypertension 40%  CAD 13.3%  CHF 13.3%  COPD 26.6%  DM 20.0%  Liver 5%  Cancer 18.3% | NR | All septic shock | 45.0% | 66.0% |
| Ghelani D et al[30] | 62.5 (16.3)  66.3 (12.9) | 58.0%  50.05 | Charlson score  1.19(1.34) & 1.20(1.30) | 22.1 (7.9) ^a^  20.6 (7.9) ^a^ | NR | 41.9% | NR |
| Haraldsen P et al[21] |  |  |  |  |  | 28.0% | NR |
| Hedlund JU et al[43] | 60.3 (18-102) | 43.0% | COPD 14.0%  Alcoholism 7.0%  Heart failure 17.0%  DM 6.0%  Cirrhosis 1.0%  Cancer 6.0% | NR | NR | 4.0% | NR |
| Hsu JL et al[84] | 69.2 (12.9)  70.3 (12.3) | 97.0%  97.4% | Charlson index>=89.2% and 94.9% of cohorts | ICU admission  15.2% and 19.3% | NR | CAP 5.0%  HCAP 9.9% | 21.2%  40.9% |
| Hynninen M et al[86] | 63 (41-76) | 52.0% | CAD 3.6%  Hypertension 20%  DM 4.9%  Liver disease 2.5%  COPD 8.0% | 8 (4-14) ^a^ | NR | 19.0% | 23.0% |
| Iwashyna TJ et al[11] | 76.9 (8.8) | 45.1% | Dialysed 4.3% | 43.2% used critical care | NR | 1996: 28.5%  2008: 15.8% | NR |
| Iwashyna TJ et al [24] sp | 73 (68-79)  73 (68-80) | 41.0%  43.0% | Not extractable | NR | NR | NR | NR |
| Jacobsson G et al[53] | 65 (NR) | NR | Major comorbidity 23.9% | NR | S: 2.8 – 20.6%  SS: 54.0 – 68.0% | 54.0% | 68.0% |
| Johnstone J et al[52] | 68.9 (17.9) | 53.05 | Nursing home resident 19.0% | PSI class IV or V 63.0% | NR | 12.0% | 28.1% |
| Karlsson S et al[65, 87] | 59.6 (15.2) | 67.0% | COPD 13.6%  DM 21.5%  Hypertension 34.9%  Cancer 4.6%  Liver disease 4.8%  Dialysis 1.5% | 24.1 (9.1) ^a^ | NR | 28.3% | 40.9% |
| Koch A et al[31] | 64 (20-90) | 58.5% | DM 28.0% | 19 (3-43) ^a^ | NR | 28.7% | NR |
| Korosec-Jagodic H et al[32] | 64.4 (13.5) | 49.0% | Included within APAPCHE score | 15.5 (6.4) ^a^ | NR | 58.0% | NR |
| Laterre P F et al[66] | 59.4 (16.6)  58.9 (16.6) | 57.5% | Included within APAPCHE score | 18.2 (5.8) ^a^  18.2 (5.8) ^a^ | NR | 17.0% | 34.0% |
| Laupland KB et al[46] | 61.5 (49.8-72.8)  64.7 (50.8-74.4) | 54%  63% | Included within APAPCHE score | 30.2 (9.8) ^a^  24.6 (8.6) ^a^ | S: 23% - 36%  SK: 51% - 61% | 41.0% | 52.0% |
| Lee H et al[67] | Population categorised by age | 55.6% | DM 14.9%  Cancer 13.5%  Hypertension 23.9%  CVS disease 11.8%  Heart failure 21.1%  Respiratory 5.6% | 17.7 (17.0-18.3) ^a^ | NR | 36.2% | 44.0% |
| Leibovici L et al[36] | 72 (18-100) | 52.0% | Bedridden 3.2%  Assisted ADL 6.5%  Curtailed ADL 19.4  Full activity 71.0% | NR | NR | 26.0% | 48.0% |
| Linder A et al[37] | 58.3 (16.5) | 65.2% | Comorbidity 48.8%  CKD 6.8%  Cancer 4.6%  HIV 8.4%  Liver disease 9.3%  Heart failure 6.1%  COPD 18.0% | 24.0 (8.7) ^a^ | NR | 39.0% | 52.6% |
| Liu V et al[58] | 71.9 (15.7) | 46.9% | Charlson index 2.0 (1.5) | Direct ICU admission 27.3% | NR | 13.6% | 38.4% |
| Lopes JA et al[68] | 50.7 (18.3)  58.8 (16.4) | 65.9% | DM 15.3%  Hypertension 21.6%  CVS disease 11.0%  Cirrhosis 11.3%  HIV 16.7%  Cancer 8.0% | 18.3 (8) ^a^  27.5 (8.6) ^a^ | NR | 28.6% | 39.6% |
| Opal SM et al[69] | 65.4 (15.0)  65.8 (15.1) | 58.4% | Included within APAPCHE score | 27.2 (4.5) ^a^  27.3 (4.5) ^a^ | NR | 27.6% | 43.6% |
| Ou SY et al[76] | 69.1 (11.8) | 69.1% | Charlson index >1 in 97% | NR | NR | 26.6% | 39.5% |
| Perl et al[70] | 62 (18-91) | 55.0% | Total active comorbidity score = 7 (0-11) | 22.7 (7-44) ^a^ | NR | 32.0% | 46.8% |
| Prescott HC et al[38] | 78.5 (8.6) | 46.5% | Charlson index 2.1 (1.7) | 19.4% received ventilation | NR | 22.2% | 54.2% |
| Poulsen JB et al[47] | 59 (46-67)  64 (52-72) | 67.3% | NR | 53 (40-41) ^s^  59 (49-74) ^s^ | All septic shock | 46.0% | 53.0% |
| Puskarich M et al [71] | 58 (16)  56 (16) | 51.9% | DM 26.7%  COPD 18.6%  Cancer 14.7%  HIV 11.2%  CKD 18.6% | 5 (3) SOFA  7 (4) SOFA | NR | 19.6% | 40.7% |
| Quartin et al[39] | 61.7 (12.6) | 99.3% | DM 16.4%  COPD 16.1%  Cancer 25.0%  CVS disease 31.2%  CKD 6.2%  Liver disease 11.2%  Haematology 9.1% | NR | S: 23.0 – 46.0%  SS: 47.0 – 71.0%  SK: 57.0 – 80.0% | 49.0% | 73.0% |
| Reade MC et al. [85] | 64.0 (18.0) | 52.0% | DM 19.0%  COPD 23.6%  Cancer 7.2%  CVS disease 26.5%  CKD 2.3%  Liver 0.7%  HIV 1.7% | Severe sepsis 31.0% on admission day | NR | 1.28% | 18.6% |
| Regazzoni CJ et al[49] | 80.8 (79.7-81.9) | 48.9% | Charlson index 2.4 (2.0-2.7) | 77.1% had severe sepsis on admission | NR | 27.9% | 59.0% |
| Sasse et al[80] | 56.8 (20.8) | 58.2% | CKD 34.0%  Cancer 24.7%  HIV 25.3%  Hypertension 10.6%  DM 17.3%  Heart failure 13.0% | 24.5 ^a^ | NR | 51.0% | 71.9% |
| Seidelin JB et al[72] | 64 (52-74) | NR | NR | All ICU population | NR | 27.0% | 52.4% |
| Shapiro NI et al [73] | 59.9 (29.4) | 54.9% | CAD 8.5%  Heart failure 12.6%  COPD 11.1%  CKD 6.4%  DM 21.3%  Cancer 18.5%  HIV 5.7%  Transplant 1.8% | 25.1% had severe sepsis/septic shock | NR | 5.4% | 21.5% |
| Utzolino A et al[51] | NR | 58.5% | CAD 57.3%  Heart failure 35.2%  COPD 28.5%  CKD 48.2%  DM 24.1%  Hypertension 53.4%  Arrhythmia 24.9%  Immune 13.4% | APACHE II score>=20 in 59.7 of cohort | NR | 47.8% | 64.3% |
| Vasile VC et al[74] | 66.6 (15.2) | 56.3% | DM 32.3%  Dialysis 23.6%  Hypertension 53.1%  CAD 15.4%  Heart failure 15.0% | APACHE III 80.8 (32.0) | NR | 27.1% | 34.2% |
| Wang T et al[7] | 70 (13) | 96% | CVS 78%  Neurology 44%  Cancer 27%  DM 29%  Gastrointestinal 13%  Pulmonary 37%  Renal 26% | 21 (7) ^a^ | NR | - | 52.7% |
| Wang HE at al [50] | Age categories reported | 52.4% | Arrhythmia 13.2%  Cancer 13.5%  COPD 20.7%  CKD 21.7%  CAD 28.6%  DVT 9.2%  DM 33.8%  Hypertension 68.7%  CAD 21.1%  Obesity 62.8%  Stroke 11.0% | Severe sepsis in 73.5% | NR | 8.9% | 23.0% |
| Waterer GW et al[42] | 58.1 (18-99) | 45.1% | CVS disease 19.9%  CVD 7.9%  Renal 3.3%  Liver 1.1%  Cancer 10.4% | Ventilation in 12.3%; Shock in 5.5% | NR | 6.4% | - |
| Weycker D et al[77] | Age categories reported | 53.4% | COPD 23.5%  CAD 23.1%  DM 17.8%  Cancer 14.8%  HIV 1.3% | All severe sepsis/septic shock | NR | 21.2% | 51.4% |
| Yaw LK et al[81] | 63 (41-78) | 64.0% | Charlson index 1(1-3) | NR | NR | 22.0% | NR |
| Yealy DM et al[44] | 60 (16.4)  61 (16.1)  62 (16.0) | 55.8% | Charlson 2.6 (2.6)  Index 2.5 (2.6)  2.9 (2.6) | 20.8 (8.1) ^a^  20.6 (7.4) ^a^  20.7 (7.5) ^a^ | All septic shock patients | 19.3% | 68.2% |
| Yende S et al[40] | 74.1 (2.9) | 59.3% | Heart failure 3.2%  CAD 27.7%  DM 20.2%  Hypothyroid 10.1% | NR | NR | 12.3% | 20.8% |
| Yende S et al[8] | 67 (17-71) | 48.2% | Charlson index >=1 in 72.1% | APACHE III score 55 (17 -54) | NR | 4.6% | 20.9% |
| Yende S et al[41] | 77.4 (7) | 44.2% | COPD 31.7%  CAD 26.6%  DM 37.5%  Cancer 32.2%  CKD 10.2% | Primary population is severe sepsis | S: NR – 27.9  SS:18.5% - 40.8% | 18.5% | 40.8% |
| Zhang K et al[28] | 53.1 (17.4) | 76.2% | Charlson index >=1 in 47.6% | 18.4 (6.8) ^a^ | NR | 53.0% | NR |

**Table S5: Egger’s test for small study effects**

| Std_Eff | Coefficient | SE | t | p>|t| | 95% CI |
| --- | --- | --- | --- | --- | --- |
| slope | 0.1719 | 0.1143 | 15.04 | 0.000 | 0.1488 – 0.1950 |
| Bias | -0.2924 | 1.9711 | -0.15 | 0.883 | -4.273 – 3.688 |
| Test of H0: no small-study effects P = 0.883 | | | | | |

**Table S6: Is post-acute mortality causally linked to sepsis from studies reporting non-sepsis controls?**

| **Author** | **Is sepsis an independent risk factor for post-acute mortality?** | **Statistically significant predictor variables for post-acute mortality** | |
| --- | --- | --- | --- |
|  | **Crude mortality comparison or adjusted hazard ratios or for sepsis compared to controls** | **Hazard ratio <1** | **Hazard ratio >1** |
| **1. General population controls** | | | |
| Linder A et al[37] | Between 1 to 5 years = 4.5 (2.2-9.1)  Beyond 5 years not significant | Beyond 5 years | Higher hazard ratio in all age categories |
| Cecere LM et al[33] | SMR = 6.0 |  | SMR was higher in all age categories.  Aged ≥65 years; Health care associated pneumonia; High-risk pneumonia; Immunosuppressant drug; HIV infection and Cardiovascular disease |
| Yende S et al [2014][41] | ICU sepsis vs. unmatched and ICU sepsis vs. matched; p<0.001 in both comparisons | - | - |
| Hedlund JU et al[43] | Relative risk of death 2.0 (1.43 – 3.2) | - | Age>50 years  Airway colonisation with Gram Negative Bacteria  Albumin <30g/L |
| Quartin AA et al[39] | General population survival curve is much better than sepsis population and hospitalised controls | - | - |
| Ghelani D et al[30] | ICU sepsis 2.4 (1.8-3.2); p<0.001  Hospital infection 2.3 (2.1 -2.5); p<0.001 | - | Age; comorbidities; men; Time in years from index admission (Hazard decreases with time) |
| Waterer GW et al[42] | Pneumonia in age>41years; absence of co-morbidities was not significant |  | Age; Cerebrovascular disease; Cardiovascular disease; Altered mental state; Hematocrit less than 35% and Increasing blood glucose |
| **2. Hospitalised non-infected controls** | | | |
| Leibovici L et al[36] | Sepsis has a significant higher risk of death; p<0.001 | - | Functional class, malignancy, age, albumin, heart failure, inappropriate antibiotic therapy, heart disease, creatinine |
| Wang HE et al[50] | 1-2 year follow up period  Unadjusted model 4.97 (3.63- 6.79)  Adjusted model 2.64 (1.85-3.77) | Abdominal infections | Male; comorbidity status; race; income; tobacco use; up to 5 years of follow-up (hazard decrease with time) |
| Yende S et al [2014][41] | ICU sepsis 27.4% vs. Hospitalised 16.1%  P<0.001 | - | - |
| Quartin AA et al[39] | 0.5 to 1 year  Sepsis 1.0 (0.7 – 1.6)  Severe sepsis 1.8 (1.3 – 2.6)  Septic shock 3.1 (1.7 – 5.7)  1-2 years  Sepsis 1.7 (1.3 – 2.4)  Severe sepsis/Septic shock 1.7 (1.3 - 2.3) | - | Hazard decreases with time although it is significantly higher than 1 |
| Yende S et al [2007][40] | Pneumonia patients (single episode)  31 days to 365 days  Unadjusted 3.9 (1.8-8.7)  Adjusted 3.9 (1.6 – 9.3)  >1 year  Unadjusted 2.0 (0.8 – 5.0)  Adjusted 2.1 (0.7 – 6.1) | - | Cancer, Fracture and heart failure comorbidities tested have a significant higher adjusted hazard ratio between 31-365 days |
| Prescott HC et al[38] | Sepsis = 44.2% vs. Control= 31.4%; p<0.01 | - | - |
| Ghelani D et al[30] | 1.42 (1.24 – 1.62); p<0.001 | As highlighted above |  |
| **3. Hospitalised infected controls** | | | |
| Ghelani D et al[30] | 1.05 (0.77 – 1.43); p=0.76 | As highlighted above |  |
| Bates DW et al[34] | 1.3 (0.76 – 2.1); p=NS | - | Severity of underlying disease |
| Yende S et al [2014][41] | ICU sepsis 27.4% vs. Hospitalised infected 21.4%  P<0.001 | - | - |
| **4. ICU controls** | | | |
| Linder A et al[37] | ICU, non-septic model  1-10 years 1.46 (1.00-2.11); p=0.047  CVS model  1-10 years 1.85 (1.49-2.30); p<0.001 | Chronic heart failure (CVS model)  Gender (ICU non-septic model) | Age (both)  Any co-morbidity (CVS model)  Hospital days (CVS model)  Dialysis (ICU-non septic model) |
| Davidson TA et al[35] | Sepsis vs. Trauma 5.45 (2.05 – 14.45) | - | 1. After controlling for risk and age: HR for comorbidities = 2.9 (1.2 to 7.3).  2. After controlling for risk and comorbidities: HR for age = 1.05 (1.03-1.07) |
| Koch A et al | Sepsis = 53.7% vs. ICU control= 35.2% | - | - |
| Yende S et al [2014][41] | ICU sepsis 27.4% vs. ICU controls 17.8%  P<0.001 | - | - |
| Korosec-Jagodic H et al[32] | Post-acute 2-year mortality  Sepsis vs. trauma = 22.0% vs. 8.0%;  *P* = 0.047 | - | - |
| Regazzoni CJ et al[49] | Cumulative 1-year mortality  Sepsis vs. Control = 54.8% vs. 35.0%;  *P* = NR | - | Organ dysfunction – respiratory, neurological ad hepatic |
| Dick A et al[25] | Sepsis 1.21 (1.04 – 1.42)  Pneumonia 1.15 (1.02 – 1.30) | Females= 0.89 (0.85 – 0.94); p<0.01 | Age  First year following index admission for pneumonia and for sepsis – interaction term significant  Medicaid HR =1.22 (1.14-1.30) |
| Wang T et al[7] | Hazard ratio for sepsis = 3.61 (1.57-8.31);p=0.001 | - | Age; Neurologic; malignancy, prior infection; vasopressor use |
| Ghelani D et al[30] | 0.74 (0.54 – 1.02); p=0.06 in overall model  0.42 (0.25 – 0.71); p=0.11 in the non-sepsis cohort model | Follow-up time since index admission; Surgical patients | Age, Men, APAPCHE II score 16-46; positive blood culture |

**eFigures**

**Figure S1 Distribution of Modified Newcastle Ottawa Score in included studies (n=59)**


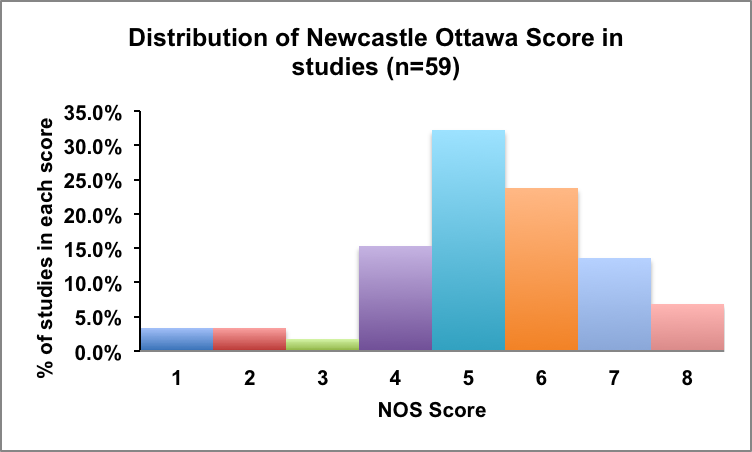

Supplement: Additional file 1: Table S1. — Search strategy. Table S2. Modified Newcastle Ottawa Score (NOS). Table S3. Description of studies included in the systematic review. Table S4. Baseline risk, acute illness risk, mortality by sepsis category, and outcomes in sepsis patients. Table S5. Egger’s Test for small-study effects. Note: data input format theta se_theta assumed. Table S6. Confounding and causality assessment from studies reporting control arms. Figure S1. Distribution of customized Newcastle Ottawa Score in the studies identified by the systematic review. (DOC 361 kb) [file 13054_2016_1276_MOESM1_ESM.doc]
